# Profile of geriatric presentations at the emergency department of a rural district hospital in South Africa

**DOI:** 10.11604/pamj.2020.36.245.22530

**Published:** 2020-08-05

**Authors:** Matthew Olukayode Abiodun Benedict, Anthonio Oladele Adefuye

**Affiliations:** 1 Department of Family Medicine, Faculty of Health Sciences, University of the Free State, PO Box 339, Bloemfontein 9300, South Africa,; 2 Division of Health Sciences Education, Office of the Dean, Faculty of Health Sciences, University of the Free State, PO Box 339, Bloemfontein 9300, South Africa

**Keywords:** Geriatric patient, Botshabelo district hospital, emergency department

## Abstract

**Introduction:**

the geriatric population is increasing in South Africa and globally. According to Statistics South Africa (STATSSA), people aged 60 years or older constitute approximately 8.4%-9.3% of the population in the Free State province, South Africa, the majority of which are rural dwellers. Elderly patients constitute a high percentage of patients presenting at the emergency department (ED) and it has been suggested that understanding the pattern of geriatric morbidities presenting at the ED can help prepare the healthcare workers and the healthcare system to confront the challenges of delivering acute geriatric care. In this present study, we compiled the profile of geriatric patients that presented at the ED of Botshabelo district hospital (BDH), Free State province, South Africa, with the aim of formulating evidence-based strategies to improving quality of service and patient outcome.

**Methods:**

this was a descriptive, retrospective cross-sectional clinical audit of all geriatric cases (≥ 65 years), that presented at the ED of BDH from January 1^st^ 2017, to March 31^st^ 2017.

**Results:**

geriatric cases accounted for 25% of the total adult ED presentation at BDH. The majority (66.6%, n=197) of the patients were female and the mean age at presentation was 75 years. The majority (63.5%) of cases were self-referred and trauma (fracture) was the most frequently diagnosed morbidity. More than half (53.7%) of the cases were classified as priority 2 (P2) and the average waiting time was 86 ± 93 minutes. Less than half of the patients (48.3%; n=143) were admitted for further management, while 36.1% (n=107) of them were discharged from the ED. The remaining 46 cases (15.5%) were referred to a tertiary hospital for further management.

**Conclusion:**

it is crucial that healthcare facilities in South Africa recognise the special needs of elderly patients due to the growing aging population. Compiling the profile of geriatric cases presenting at ED can help identify crucial area of need and help prepare the healthcare workers and the healthcare system to confront challenges of delivering acute geriatric care. Findings presented herein will assist in formulating evidence-based strategies to improve geriatric patient outcome at the ED in BDH.

## Introduction

Globally, the proportion of the populations of people aged 65 years and over are increasing at a disproportionate rate, compared to other age groups [1]. According to the WHO projections, the population of people aged 65 years or older will triple to 1.5 billion (16% of the population) by 2050 and that in about five years’ time, the number of people aged 65 years or older will outnumber the under-five age group [2]. It has been reported that the vast majority of older people and the most rapidly aging populations reside in the less developed nations [2, 3]. According to Statistics South Africa 2019 mid-year population estimate report, approximately 9% (i.e. 5.3 million) of the population is 60 years or older and the proportion of this age group (≥ 60 years) is increasing over time [4]. Similarly, people aged 60 years or older constitute approximately 8.4%-9.3% of the population in the Free State Province, South Africa, majority of which are rural dwellers [5]. Thus suggesting an increased burden on the rural health systems as a consequence of the chronic disease and frailty experienced by this age group [6].

In contrast to younger patients, elderly patients are more prone to medical conditions or ill health and thus constitute a high percentage (approximately 21%) of those presenting at the emergency department (ED) [7]. In addition, this group of patients (elderly patients) often present at the ED with atypical or complex symptoms associated with multiple comorbidities [8], that require a more comprehensive and multi-disciplinary interventions [9] Providing acute geriatric care can pose significant challenges to healthcare workers who often lack training in geriatric issues [10, 11]. Therefore, it is essential to develop and introduce strategies to improve outcomes in older patients in the ED [8]. It has been suggested that understanding the pattern of geriatric morbidities presenting at the ED can help prepare the healthcare workers and the healthcare system to confront the challenges of delivering acute geriatric care [12]. Hence in this present study we compiled the profile of geriatric cases presenting at the ED of a rural district hospital (Botshabelo district hospital) in the Free State province, South Africa. It is our assumption that findings by this study will help develop appropriate strategies and instruments, which can be utilized to support the clinical decision matrix and improve the outcome of geriatric cases presenting at the ED of Botshabelo district hospital (BDH).

## Methods

This was a descriptive, retrospective cross-sectional clinical audit of all geriatric cases (≥ 65 years), that presented at the ED of BDH from January 1^st^ 2017, to March 31^st^ 2017. This time (January-March) was selected to minimize the impact of seasonal variation in ED attendances.

**Study setting:** Botshabelo District Hospital (BDH) is a 135 bed government-funded hospital in Botshabelo, a community of about 210,000 people. It is one of the three district hospitals in Mangaung metropolitan municipality in the Free State province. The hospital serves as the referral centre for the 13 Primary health clinics in Botshabelo community as well as Theunissen and Verkeerdevlei clinics. It is about 57Km away from Bloemfontein where its referral regional and tertiary institutions are located. The ED at BDH has 12 beds (adult beds=10, paediatrics beds=2) and see up to an average of 56 ED cases per day (43 adults, 13 children).

**Data collection:** particulars of geriatric patients (≥ 65 years), that presented at BDH ED from 1^st^ January 2017 to 31^st^ March 2017 were retrieved from the ED register. The applicable case records were subsequently retrieved from the records department. Relevant information such as demographic details, time/day of visit, mode of referral, waiting time, diagnosis, outcome, and rank of attending doctor were captured using the datasheet designed by one of the researchers (MB), based on trends observed in similar studies [12].

**Data analysis:** data were entered into an excel spreadsheet (Microsoft Office Professional Plus 2016) and analyzed using the Statistical Package for Social Sciences (IBM SPSS Statistics 25). Results were summarized by descriptive statistics for continuous data and frequencies and percentages for categorical data.

**Ethical aspects:** the Health Sciences Research Ethics Committee (HSREC), University of the Free State (UFS-HSD2019/0897/708), approved this study. The data retrieved from the patient case files for the purpose of this study were handled confidentially.

## Results

In total, 324 geriatric cases presented at the emergency department of BDH over the 3-month study period, accounting for approximately 25% of the total adult ED presentation at BDH [i.e. 324/ (1290x100)]. Only 296 (91.3%) of the 324 case files reviewed, had sufficient information and were included in the study. The remaining 28 patient files were either missing or had incomplete information and could not be analyzed.

**Demographic profile:** the majority (66.6%, n=197) of the patients were female, while male patients made up 33.4% (n=99) of the cases. The majority, that is n=87 (29.4%) were within the age group 65-70 years, while the mean age at presentation was 75 years.

**Timing of visit:** daytime presentation (8: 00 am- 4: 00 pm) was 50.7% (n=150) while after hours (beyond 4: 00pm) presentation was 49.3% (n=146). The majority (78.4%; n=232) of the cases presented during the weekdays (i.e. Monday-Friday), while only 64 cases (21.6%) presented during weekends (i.e. Saturday-Sunday).

**Mode of referral:** one hundred and eighty-eight cases (63.5%) presented as self-referral, 24.7% (n=73) were referred from the peripheral clinics, 3.4% (n=10) were referred from private practices, while 8.4% (n=25) were down referral.

**Triage acuity:** the majority (53.7%; n=159) were triaged as P2 (Priority 2; Moderate to serious injury/illness, not immediately life threatening), 29.1% (n=86) were triaged as P1 (Priority 1; patient has an acutely life-threatening illness or injury and is unstable), while 17.2% (n=51) were P3 (Priority 3; patient does not have potentially life-threatening illness). There were no cases of P4 and P5 seen during the study period.

**Waiting time:** the Mean ± SD waiting time (i.e. post triage to end of doctor’s consultation) was 86 ± 93 minutes (min=3 minutes; Maximum=590 minutes).

**Attending doctor at BDH:** the majority (54.1%; n=160) of the cases were attended to by community service doctors (CSDs) (Table 1).

**Table 1:** cadre and number of geriatric cases seen by doctors at BDH

Cadre	Number of geriatric cases n (%)
Community service medical officer	160 (54.1)
Grade 1 medical officer	48 (16.2)
Grade 2 medical officer	25 (8.4)
Grade 3 medical officer	17 (5.4)
Registrar (Family Med)	46 (15.5)

**Figure 1:**
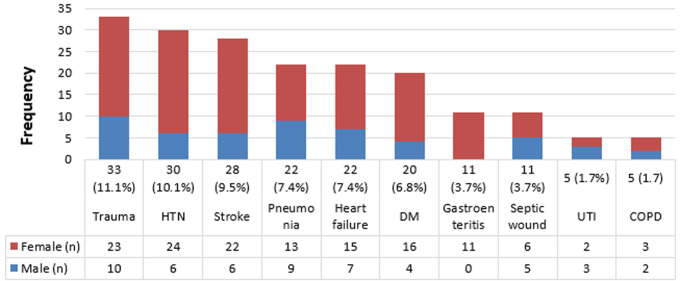
most frequent diagnosis among geriatric patients presenting at BDH ED

**Most frequent diagnosis:** data presented in Figure 1 shows that trauma in the elderly was the most frequent diagnosis made (11.1%; n=33) and majority of these cases were seen in female patients (n=23 females vs n=11 males). Further findings reveal that 54.5% (n=18) of the trauma cases seen were fractures, 21.2% (n=7) were soft tissue injuries, while only 12.1% (n=4) were limb dislocations. Head injury (n=1), eye injury (n=1), and chin laceration (n=1) made up the remainder of the trauma cases.

**Patient outcome:** findings obtained by this study reveal that the majority (48.3%; n=143) of the patients were admitted in for further management, while 36.1% (n=107) of them were discharged from the ED. Only n=46 (15.5%) of the cases were referred to a tertiary hospital for further management.

## Discussion

Apt and Grieco (1994), reported that the elderly population across Africa is evenly distributed [13] and the Southern African region has the highest percentage [3]. In South Africa, the elderly population has increased from 6.7% in 1996 to 9% (i.e. 5.3 million) of the population in 2019 [4, 14] and it has been estimated to rise to 10.4% by 2025 [15]. It is therefore of necessity that all healthcare facilities across the country make provision for the care of these group of patients, since it has been shown that the prevailing disease patterns within these age group vary from that of the younger generation [12]. In addition, geriatric patients are four to six times more likely to be admitted to an emergency unit than a non-elderly patient [16]. There is an urgent need for healthcare facilities to profile the elderly patients presenting at their emergency department (ED) in order for adequate training to be implemented and for appropriate strategies and instruments to be put in place [14]. Management of geriatric patients in the ED is a vital issue, not only because of their rapidly increasing population, but also because of their specific and complex characteristics and risk of adverse outcomes [17]. In this present study, we compiled the profile of geriatric patients that presented at the ED of BDH in the Free State province, South Africa, over a 3-month period (1^st^ January 2017 to 31^st^ March 2017) with the aim of formulating evidence-based strategies to improve geriatric patient outcome at the ED in BDH.

Findings by this study reveal that geriatric patient constitute 25% of the total adult presentation at BDH ED during the study period corroborating findings by other studies that have reported that geriatric patients are increasingly accessing EDs and the number of geriatric patient admitted to EDs is growing [18, 19]. In a study carried out to compile the profile of geriatric patient in an ED in the Netherlands, Schrijver *et al*. (2013) reports that only 35% of their participants were male [20]. Similarly, findings presented herein reveal that female geriatric patients constitute the majority (66.6%) of the cases seen at BDH ED. This findings is consistent with results from other similar studies [21-25], thus, suggesting that female geriatric patients are frequent users of the ED compared to their male counterpart. Our findings reveal that there was no significant difference in time of visit to the ED at BDH (i.e. daytime presentation vs afterhours presentation). This is contrary to prior findings that reported an increase in presentation during evening or night shifts [25, 26]. Patients with morbidities whose symptoms usually disturb during the night will often present during afterhours [25], while patient with emergencies such as trauma often present immediately at the ED regardless of the time of occurrence. We observed that trauma, and in particular, fractures constitute the majority of the diagnosis made (Figure 1). Moreover, the majority of the patients were triaged at higher acuity (urgent priority, P1=29.1% and semi-urgent priority, P2=53.7%). It is therefore probable that the variance in pattern of disease presentation can determine the time of presentation at ED.

In this present study, we observed that the majority of the geriatric patients who presented at BDH ED were self-referred. Numerous factors such as the need for afterhours care [27], getting help faster [28], perceived superior treatment at hospitals [29], lack of access to other care [30], and a belief that the problem was serious enough to warrant emergency treatment [30] has been associated with the reason why patients bypass the primary health care clinics and go directly to the ED. It is very likely that one or more of the aforementioned factors might have influenced the pattern of self-referral seen in this study. Inappropriate self-referrals to the ED has been reported to impose cumulative strain on the ED system with consequent negative effects on the quality of patient care [28, 31]. It is very plausible that incessant self-referral might impose needless burden on the ED system at BDH leading to poor quality of care for the geriatric age group. The waiting time in emergency department has been suggested as one of the key indicator in measuring the quality of hospital services [32]. Findings by this study reveal that the Mean ± SD waiting time post triage to the end of doctor’s consultation was 86 ± 93 minutes. This is more than the national standard [33], and suggest poor hospital service. Prolonged waiting time may be due to inexperience of doctors (mostly junior doctors, just post-internship) attending to these patients (Table 1). Lengthy waiting time in the emergency room may hinder services to other patients requiring emergency medical care and cause patient dissatisfaction [34-36]. Therefore, we recommend that the hospital management at BDH develop strategies to deliver quality services at the right time and reduce the patients’ waiting time at ED.

The findings by this study, which show that accidental injuries (traumas) were the most common reason for visiting the ED is consistent with findings by prior studies [12, 23, 37, 38]. Other studies have reported disorders related to the circulatory and respiratory systems as the most common presenting reasons for the ED visits [25]. Fractures in the elderly is an important public health issue, particularly as incidence increases with age, and the population of the elderly age group is growing [39]. The prevalence of osteoporosis and the risk of fracture are higher in postmenopausal women than in older men and this is partially attributed to differences in bone mineral density (BMD), bone size, and bone strength between men and women [40]. Similarly, we observed that fractures constitute the majority (54.5%; n=18) of trauma cases presenting at BDH ED and this was found to be more in females than in males (Figure 1). This is consistent with findings by Ukkonen *et al*. (2019) [41]. Further findings by this present study, which show that 48.3% (n=143) and 36.1% (n=107) of the patients were admitted for further management and discharged from the ED, respectively suggests that the levels of medical conditions of the majority of elderly patients who visited the BDH ED were mild to moderate. A major limitation of this present study is that it was conducted during one season (summer months) hence, the effect of seasonal variation on disease profile or ED visit could not be ascertained.

## Conclusion

It is crucial that healthcare facilities in South Africa recognise the special needs of elderly patients due to the growing ageing population. Compiling the profile of geriatric cases presenting at ED can help identify crucial area of need and help prepare the healthcare workers and the healthcare system to confront challenges of delivering acute geriatric care. Findings presented herein will assist in formulating evidence-based strategies to improve geriatric patient outcome at the ED in BDH.

### What is known about this topic

- Geriatric population is increasing in South Africa and globally;
- Elderly patients constitute a high percentage of patients presenting at the emergency department;
- Elderly patients often present at the ED with atypical or complex symptoms associated with multiple comorbidities, making acute geriatric complex.

### What this study adds

- This study confirmed that geriatric age group constitute a high percentage of patients presenting at the BDH emergency department;
- This present study contextualised the profile of elderly patients presenting at BDH emergency department during summer;
- This study provides data that will inform evidence-based strategies to improve geriatric patient outcome at the ED in BDH.
